# Characteristics and nutrient function of intestinal bacterial communities in black soldier fly (*Hermetia illucens* L.) larvae in livestock manure conversion

**DOI:** 10.1111/1751-7915.13595

**Published:** 2020-05-25

**Authors:** Yue Ao, Chongrui Yang, Shengchen Wang, Qingyi Hu, Li Yi, Jibin Zhang, Ziniu Yu, Minmin Cai, Chan Yu

**Affiliations:** ^1^ State Key Laboratory of Biocatalysis and Enzyme Engineering School of Life Sciences Hubei Engineering Research Center for Bio‐enzyme Catalysis Hubei University Wuhan China; ^2^ State Key Laboratory of Agricultural Microbiology College of Life Science and Technology National Engineering Research Centre of Microbial Pesticides Huazhong Agricultural University Wuhan China

## Abstract

The potential utility of black soldier fly larvae (BSFL) to convert animal waste into harvested protein or lipid sources for feeding animal or producing biodiesel provides a new strategy for agricultural waste management. In this study, the taxonomic structure and potential metabolic and nutrient functions of the intestinal bacterial communities of BSFL were investigated in chicken and swine manure conversion systems. Proteobacteria, Firmicutes and Bacteroidetes were the dominant phyla in the BSFL gut in both the swine and chicken manure systems. After the larvae were fed manure, the proportion of Proteobacteria in their gut significantly decreased, while that of Bacteroidetes remarkably increased. Compared with the original intestinal bacterial community, approximately 90 and 109 new genera were observed in the BSFL gut during chicken and swine manure conversion, and at least half of the initial intestinal genera found remained in the gut during manure conversion. This result may be due to the presence of specialized crypts or paunches that promote microbial persistence and bacteria–host interactions. Ten core genera were found in all 21 samples, and the top three phyla among all of the communities in terms of relative abundance were *Proteobacteria, Firmicutes and Bacteroidetes*. The nutrient elements (OM, TN, TP, TK and CF) of manure may partly affect the succession of gut bacterial communities with one another, while TN and CF are strongly positively correlated with the relative abundance of *Providencia*. Some bacterial taxa with the reported ability to synthesize amino acids, *Rhizobiales*, *Burkholderia*, Bacteroidales, *etc*., were also observed in the BSFL gut. Functional analysis based on genes showed that intestinal microbes potentially contribute to the nutrition of BSFL and the high‐level amino acid metabolism may partly explain the biological mechanisms of protein accumulation in the BSFL body. These results are helpful in understanding the biological mechanisms of high‐efficiency nutrient conversion in BSFL associated with intestinal microbes.

## Graphical abstract

The potential utility of black soldier fly larvae (BSFL) to convert animal waste into harvested protein or lipid sources provides a new strategy for agricultural waste management. In this study, the taxonomic structure and potential metabolic and nutrient functions of the intestinal bacterial communities of BSFL were investigated in chicken and swine manure conversion systems. The results are helpful in understanding the biological mechanisms of high‐efficiency nutrient conversion in BSFL associated with intestinal microbes.

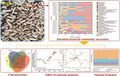

## Introduction

To control the pollution caused by the increasing amount of industrial livestock and poultry manure waste (Bai *et al*., 2016; Qian *et al*., 2018) and allow nutrient recycling (Burkholder *et al*., 2007) for agricultural applications, researchers have developed manure treatment technology by using the black soldier fly larvae (BSFL, *Hermetia illucens* L.; Diptera: Stratiomyidae). BSFL can efficiently convert animal manure into protein and fat suitable for feeds; this strategy is highly efficient and low cost (Sheppard *et al*., 1994; Lalander *et al*., 2013; Čičková *et al*., 2015). In addition, pollutants excreted with animal waste, such as pathogens (Greger and Koneswaran, 2010), veterinary antimicrobials (Campagnolo *et al*., 2002) and heavy metals (Song *et al*., 2014), could be rapidly and effectively reduced by BSFL to mitigate their risk to the environment and human health (Lalander *et al*., 2016; Cai *et al*., 2018a,b,c).

The promising ability of BSFL to convert animal waste is encouraging. However, there is little research on the taxonomic identities and potential functions of BSFL gut microbiome in waste conversion. The gut microbiota of many insects greatly expands the physiological capabilities of insects by contributing to their nutrition, protection against parasites and pathogens, modulation of immune responses and communication (Engel and Moran, 2013). The strong cellulose degradation ability of gut microbiota, for example, supports termites (Warnecke *et al*., 2007). Moreover, the gut microbial community has been observed to help mealworms achieve rapid polystyrene degradation (Yang *et al*., 2015). The BSFL intestinal bacterial communities played critical roles for larvae to degrade tetracycline rapidly (Cai *et al*., 2018a,b,c). Bacterial genes encoding enzymes in the BSFL gut, such as proteases, cellulases and lipases, can hydrolyse starch, cellulose, proteins and lipids, and can thereby contribute to the decomposition and recycling of biological waste and other accumulated nutrients in insect hosts (Jeon *et al*., 2011; Lee *et al*., 2014). As such, investigating the intestinal microbiota may be a feasible way to understand the biological mechanisms of high‐efficiency nutrient conversion in BSFL.

In this study, we added BSFL to chicken and swine manure and documented changes in the microbial community and nutrient profiles over time with the aim of evaluating the structures of the relevant larval intestinal bacterial communities and their function in nutrient metabolism. Hence, the objectives of this study include (i) characterization of the dominant bacterial communities in the BSFL gut, (ii) determination of the correlations between changes in nutrient profile and changes in microbial community composition, and (iii) functional assessment of the potential contributions of intestinal bacteria to nutrition and other beneficial behaviours in BSFL.

## Results and discussion

### Analysis of bacterial 16S rRNA gene sequences

**Fig. 1:**
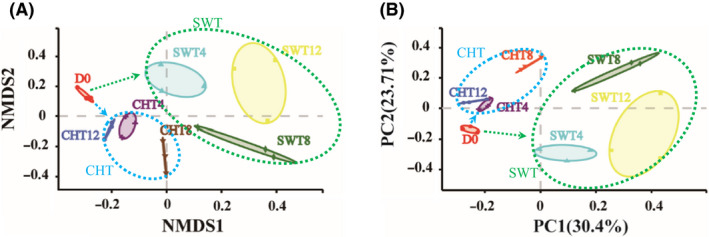
Non‐metric multidimensional scaling analysis (NMDS, A) and principal coordinates analysis based on the Bray–Curtis distances (PCoA, B) of the 21 BSFL intestinal microbe samples at the OTU level. D0: for the initial for the initial larval gut; CHT and SWT: for the chicken manure and swine manure treatment systems; 0/4/8/12: sampling day. The percentages on the PCoA axes indicate how much of bacterial community is explained.

A total of 1 224 739 high‐quality 16S rRNA sequence reads were obtained from 21 samples and identified as belonging to bacteria (Table S1). The average 16S rRNA gene sequence length was 446 nt. Rarefaction curves based on OTUs at a nucleotide similarity level of 97% suggested that the majority of bacterial phylotypes in the BSFL gut were included in this study (Fig. S1). Non‐metric multidimensional scaling analysis (NMDS) and principal coordinate analysis (PCoA) showed that the larval gut samples for each group and manure condition clustered well (Fig. 1). The points (SWT) representing the larval gut samples from swine manure clustered separating from those (CHT) from chicken manure, which suggests that the larval gut microbes develop as two different bacterial communities in chicken and swine manure, consistent with previous reports (Jeon *et al*., 2011; Colman *et al*., 2012; Yun *et al*., 2014). It also should be noted that clearing the gut prior to sequencing could also have impacted the detected BSF microbial community and some of the functionality.

### BSFL intestinal bacterial communities in the manure conversion systems

**Fig. 2:**
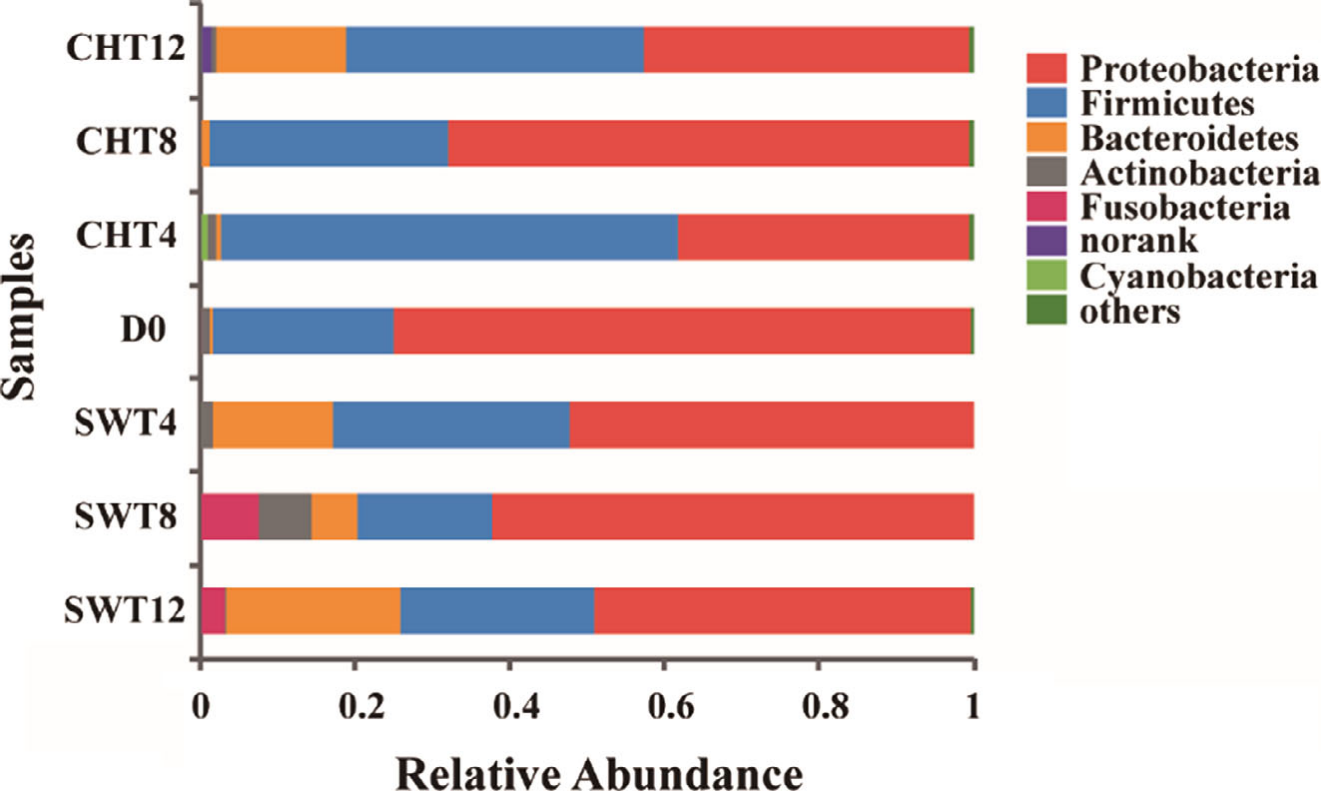
The average relative abundance of phylum‐level taxa for BSFL intestinal bacterial in chicken manure (CHT) and swine manure (SWT) treatment systems (*n* = 3). D0: for the initial larval gut; CHT/SWT 4/8/12: for the larval gut from manure system in the 4th/8th/12th day. More details of phylum‐level taxa for 21 samples are shown in Fig. S2.

Proteobacteria, Firmicutes and Bacteroidetes were the dominant phyla (Figs 2 and S2) in the larval gut, consistent with the main taxa of the intestinal microbes of insects (Zurek *et al*., 2000; Su *et al*., 2010; Jeon *et al*., 2011; Zheng *et al*., 2013; Singh *et al*., 2015; Boccazzi *et al*., 2017; Scully *et al*., 2017; Wang *et al*., 2017). Amplicons derived from Proteobacteria comprised 74.5% of the sequenced amplicons in the larval gut (D0); this relative abundance declined to 48.8% and 42.0% in swine and chicken manure, respectively, after 12 days. The relative abundance of amplicons derived from Firmicutes population (23.5% for D0) was maintained and displayed a dynamic and relative process (17.5–30.7% for SWT 4/8/12) in the BSFL gut in swine manure, but increased (30.8–59.3% for CHT 4/8/12) by 1.3‐ to 2.5‐fold in chicken manure. Interestingly, Bacteroidetes was observed at very low proportions (0.48%) at D0 but accounted for 16.71% and 22.50%, respectively, of the total bacteria in the chicken and swine manure conversion systems, especially after 12 days. Some studies indicate that increases in Bacteroidetes may be related to antibiotic degradation in organic waste treatment systems with housefly larvae or BSFL (Zhang *et al*., 2014; Cai *et al*., 2018a,b,c). In fact, chicken and swine manure in China commonly contain antibiotic residues (Wu *et al*., 2011; Cho *et al*., 2012), which may explain why Bacteroidetes increases in larval gut.

**Fig. 3:**
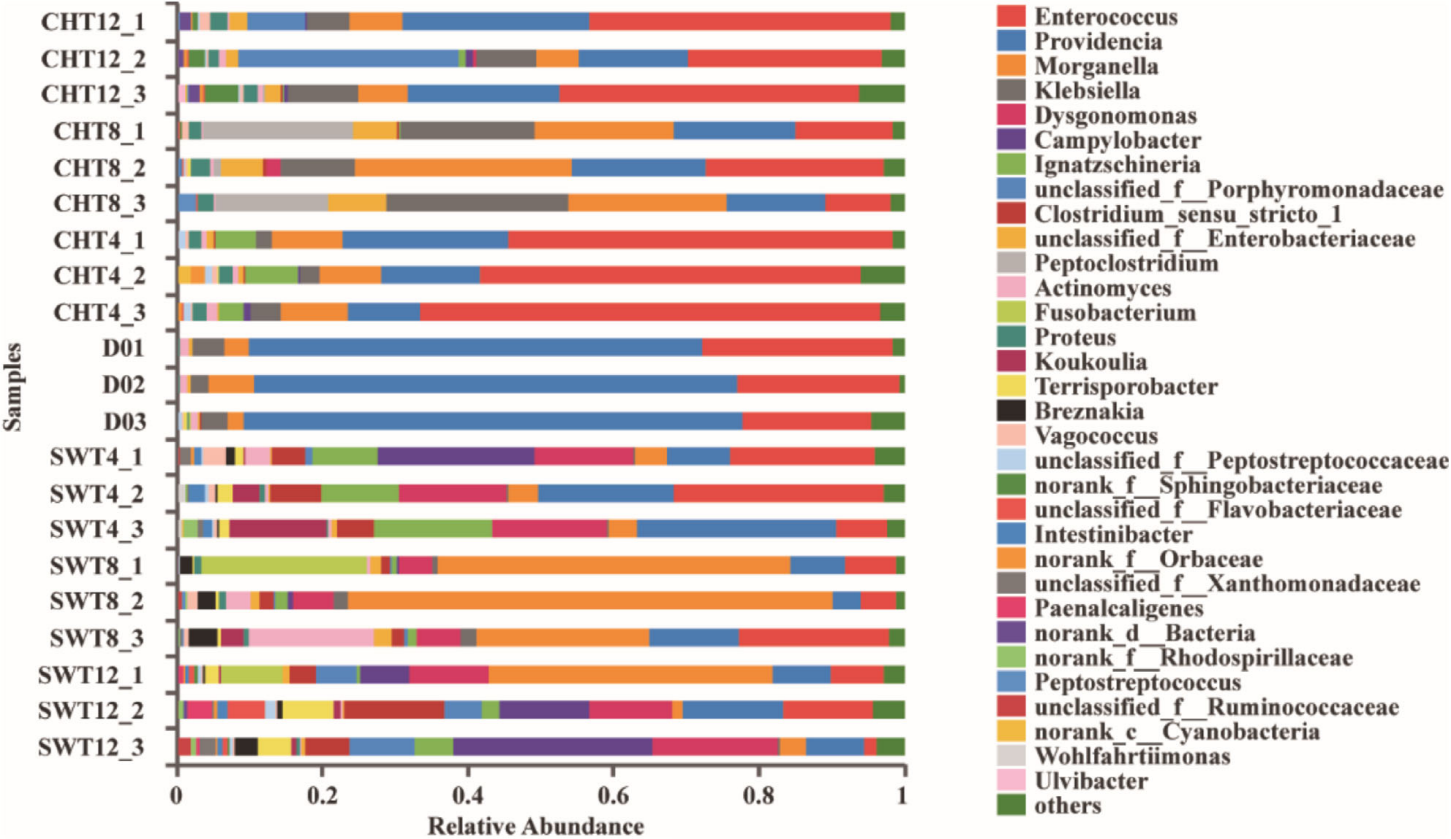
The relative abundance of genus‐level taxa for BSFL intestinal bacterial in chicken manure (CHT) and swine manure (SWT) treatment systems. Unclassified: the OTU could not be reliably assigned to genus or species level; no_Rank: there is no clear classification information or classification name at a certain classification level (based on the database).

The larval intestinal bacterial community structure is complex at the genus level (Fig. 3). The dominant genera of BSFL gut microbes showed remarkable variability during conversion of chicken and swine manure and their relative abundances differed over time. *Providencia* and *Enterococcus* accounted for 87.91% of the BSFL intestinal bacterial community at D0. After 12 days, the proportion of *Providencia* (65.84 ± 3.16%) decreased to 20.50 ± 5.47% and 9.97 ± 3.37% in chicken and swine manure systems, respectively, while *Enterococcus* (22.07 ± 4.24%) decreased to 7.04 ± 5.38% in swine manure system but increased to 36.54 ± 8.36% in chicken manure system. *Campylobacter* (6.83–27.43%), *Morganella* (1.41–39.21%), *Dysgonomonas* (10.80–17.34%) and *Providencia* (8.00–13.86%) made up the dominant genera of the BSFL intestinal bacterial community in swine manure systems, while *Providencia* (14.88–25.81%) and *Enterococcus* (26.89–41.38%) made up those in chicken manure systems. These variations may be attributed to the effects of nutrition and indigenous microorganisms in manure (Jeon *et al*., 2011; Colman *et al*., 2012; Yun *et al*., 2014).

**Fig. 4:**
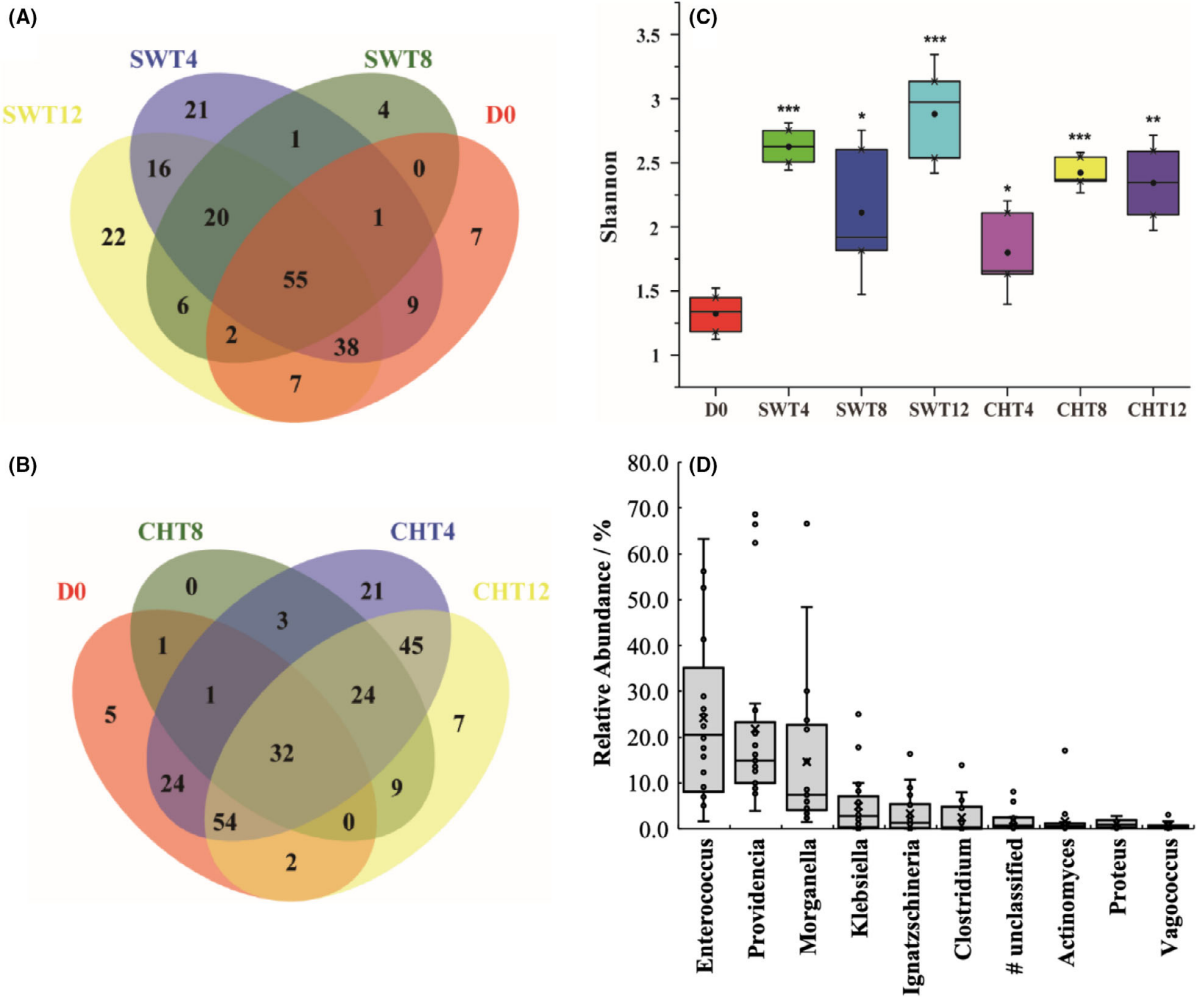
(A,B) Venn diagram of the unique and shared OTUs of microbes in the gut of BSFL in swine manure (SWT, A) and chicken manure (CHT, B) treatment systems; (C) Shannon indices for each group sample (*n* = 3); and (D) relative abundances of the 10 genera (core genera) existing in all samples (*n* = 21). The box plots denote 25th to 75th percentile; horizontal line: median; whiskers: 10th and 90th percentile; and ×: mean. ^*^: 0.01 < *P* ≤ 0.05; ^**^: 0.001 < *P* ≤ 0.01; ^***^: *P* ≤ 0.001, compared to D0. ^#^: unclassified in Enterobacteriaceae.

Compared to D0, approximately 90 and 109 new genera were observed in the BSFL gut during the respective conversions of swine and chicken manure (Fig. 4A and B). Meanwhile, the Shannon indices of the intestinal microbes in gut samples obtained after conversion (SWT 4/8/12 and CHT 4/8/12) were significantly higher than those in D0 (Fig. 4C). These results indicated that the population diversity of intestinal microbes was increasing during the manure conversion. These new intestinal genera may originate from the food (i.e. manure) of BSFL.

Interestingly, a great number of intestinal genera that were observed at initial BSFL gut (D0) were also observed in the larval gut in the swine and chicken manure systems, which ranged from 61.04% to 65.2% and from 48.6% to 54.4% respectively. Thus, at least half of the initial intestinal genera observed appeared to be consistently maintained in the gut, which may be due to the presence of specialized crypts or paunches that promote microbial persistence and bacteria–host interactions (Engel and Moran, 2013). Moreover, we also analysed the common genera among the 21 BSFL gut samples and found 10 genera existing in all samples (Fig. 4D): *Enterococcus* (the average relative abundance of 24.1%), *Providencia* (21.7%), *Morganella* (14.5%), *Klebsiella* (4.9%), *Ignatzschineria* (3.3%), *Clostridium* (2.4%), unclassified in Enterobacteriaceae (1.8%), *Actinomyces* (1.6%), *Proteus* (1.1%) and *Vagococcus* (0.6%). However, bacterial taxa with low abundance could also play disproportionately large roles in niches and functions (Van Goethem *et al*., 2017; Sun *et al*., 2018).

### Effect of nutrient elements on the BSFL intestinal bacterial community structure

**Fig. 5:**
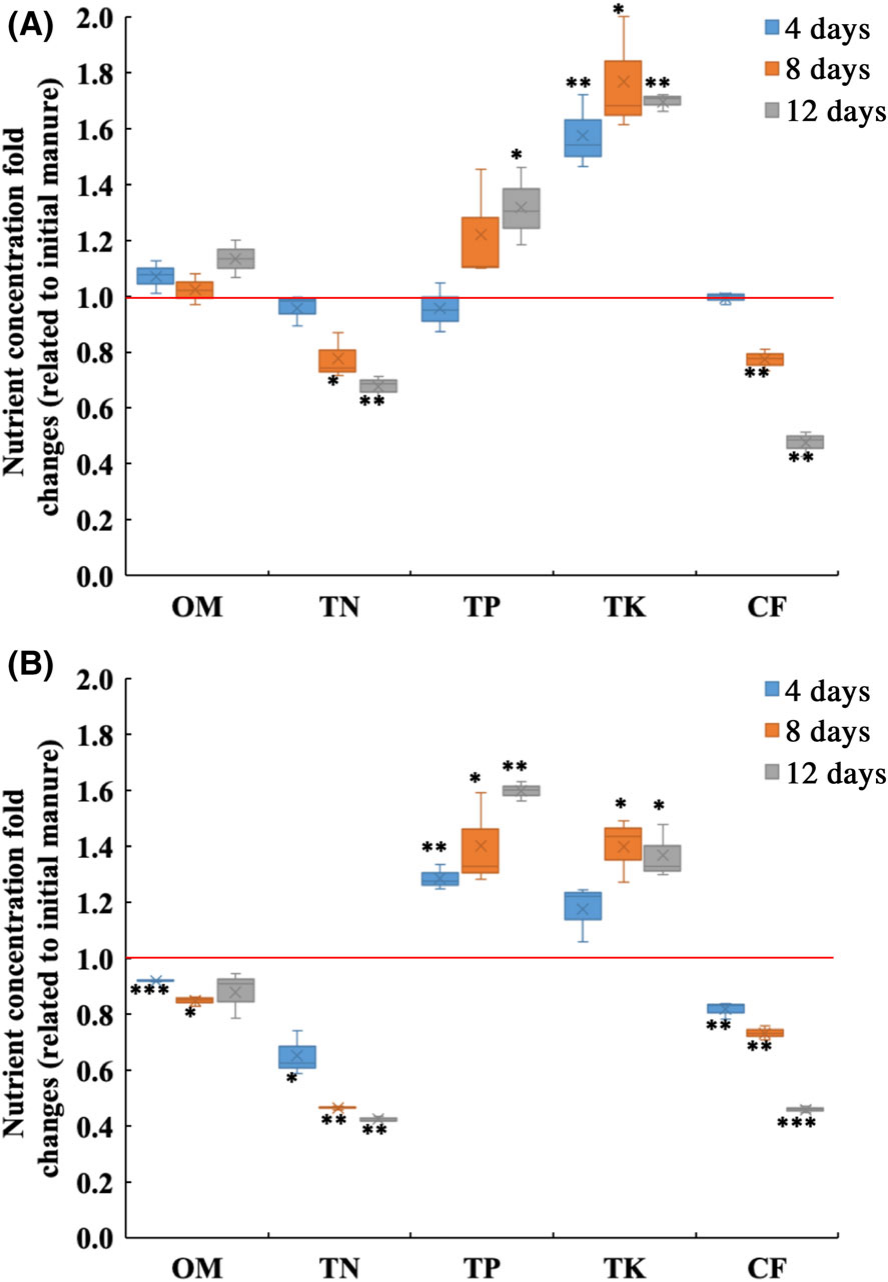
The fold changes (related to initial manure) of nutrient concentration (*n* = 3) in (A) swine manure and (B) chicken manure treatment systems. OM: organic matter; TN: total nitrogen; TP: total phosphorus; TK total potassium; and CF: crude fat. The box plots denote 25th to 75th percentile; horizontal line: median; whiskers: 10th and 90th percentile; and ×: mean. ^*^: 0.01 < *P* ≤ 0.05; ^**^: 0.001 < *P* ≤ 0.01; ^***^: *P* ≤ 0.001, according to Student’s *t*‐test.

**Fig. 6:**
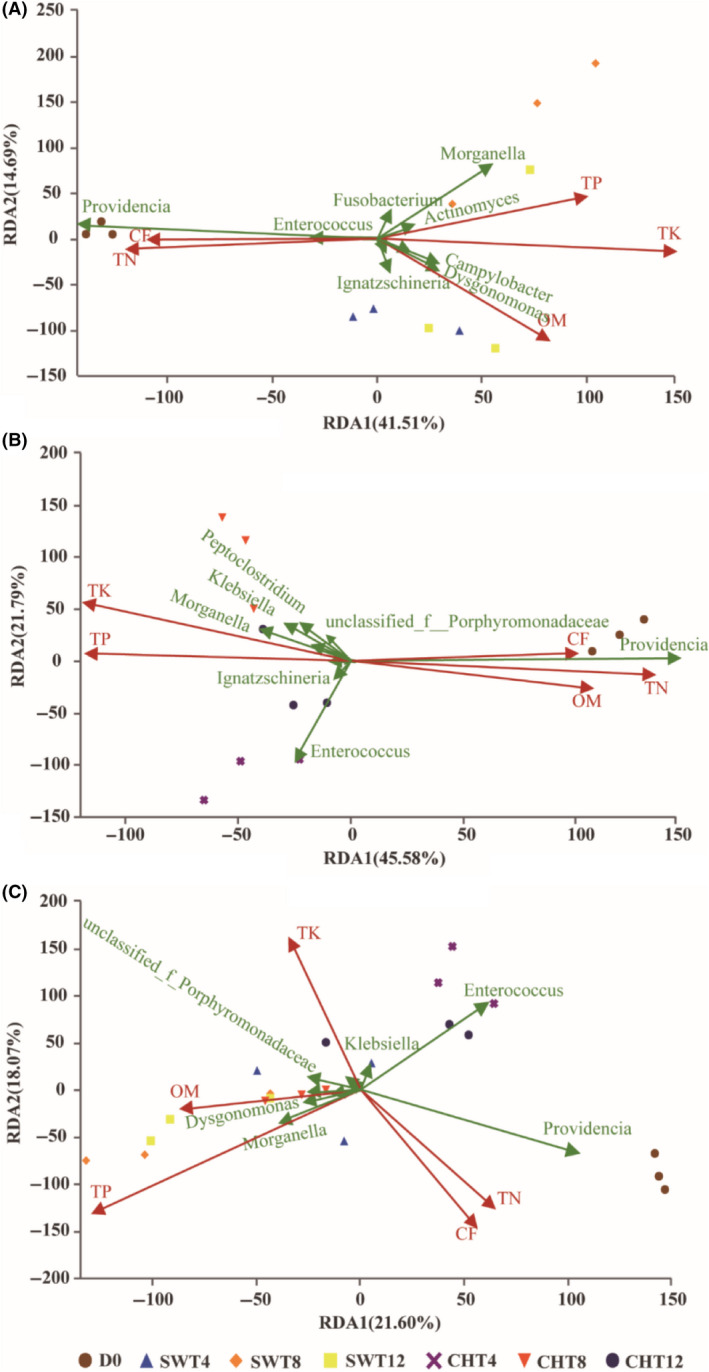
Redundancy analysis (RDA) of nutritional variables (OM, TN, TP, TK and CF) and intestinal genus communities in swine manure system (A, *n* = 12), chicken manure system (B, *n* = 12) and both system (C, *n* = 21), based on the concentration of environmental variables and the relative abundance of top 10 genera, using the vegan‐package in R project with the original data of the same unit for nutritional variables.

To determine whether changes in nutrient profiles (i.e. OM, TN, TP, TK and CF) correlated with changes in the microbial community, we evaluated these parameters (Fig. 5) and carried out redundancy analysis (RDA, Fig. 6). After 12 days, the concentrations of OM, TP and TK increased by 13.5%, 31.8% and 69.7%, respectively, while those of TN and CF decreased by 32.2% and 52.3%, respectively, in swine manure relative to initial levels. In chicken manure, the concentrations of TP and TK increased by 59.9% and 36.9%, respectively, while those of OM, TN and CF decreased by 12.0%, 57.6% and 54.0%, respectively, relative to initial levels.

The RDA results indicate that OM, TN, TP, TK and CF are closely related to the bacterial community structure of manure. In the swine manure system (PDA 1 and 2 explaining total 56.20% of nutritional variables), *Providencia* and *Enterococcus* were strong positively related to CF and TN; similarly, *Morganella* was positively related to TP. By comparison, in the chicken manure system (PDA 1 and 2 explaining total 67.37% of nutritional variables), *Providencia* was strong positively related to OM, TN and CF, while *Morganella* was positively related to TK and TP. However, *Enterococcus* was not clearly related to any of these nutrient elements. These differences suggest that other factors, such as antibiotic residues, heavy metals, environmental bacteria, clearing the gut prior to sequencing and bacteria–host interactions, may affect the development of the BSFL intestinal bacterial community.

However, RDA of all 21 samples in chicken manure and swine manure (PDA 1 and 2 explaining total 39.67% of nutritional variables) indicated that TN and CF are positively correlated with the relative abundance of *Providencia*, one dominant genera of the BSFL intestinal bacterial community. TN is a key nutritional element for bacterial growth (Ogunwande *et al*., 2008; Gao *et al*., 2010). Fat (CF) is an important means of stored energy for BSFL, while biowaste lipids could be decomposed in the larval gut into free fatty acids or mono‐ and diglycerides for absorption by gut cells and use in larval metabolism (Kim *et al*., 2011; Carvalho *et al*., 2012). Thus, microbes from the genus *Providencia* might be important for lipid and protein conversion in the gut.

### Functional prediction of the intestinal microbiota

Intestinal bacteria contribute to the nutrition of insects (Engel and Moran, 2013); indeed, bacterial cells are important nutrient sources for insects. High‐level lysozyme expression promotes bacterial digestion and nutrient release in the gut of *Drosophila* (Daffre *et al*., 1994; Miller *et al*., 2008). The intestinal bacterial communities in the BSFL gut maintain a dynamic balance, and bacteria and other microorganisms ingested with manure might be an important source for larvae.

Plant‐associated Rhizobiales are well known for their ability to fix nitrogen (Masson‐Boivin *et al*., 2009). *Burkholderia* supplies essential amino acids and vitamins to its alydid stinkbug host, *Riptortus pedestris*. (Kikuchi *et al*., 2007). The endosymbiont of termite gut protists, CfPT1‐2, which belongs to the order Bacteroidales, has functional genes streamlined for the production of amino acids and cofactors, nitrogen fixation, and ammonium, urease and urea transport (Hongoh *et al*., 2008a,b). In this study, Rhizobiales, *Burkholderia* and Bacteroidales were found in the BSFL gut with average proportions (referring to all samples) of 0.05%, 0.42% and 7.37% respectively. Thus, these bacteria may have similar nutritional roles in the BSFL gut.

**Fig. 7:**
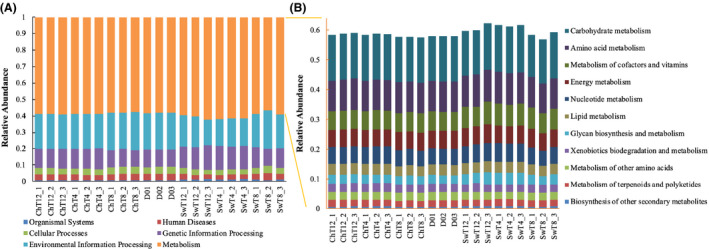
KEGG (Kyoto Encyclopedia of Genes and Genomes) pathway functional classifications (A: for all functions observed; B: for metabolism functions) of intestinal bacterial of BSFL in chicken and swine manure treatment systems (21 samples).

At the gene level, although the BSFL intestinal bacterial community structures significantly differed over time in the chicken and swine manure treatment systems, their predicted metabolic functions were similar to what was observed in insects from the D0 diet (Fig. 7), and accounted to 56.9–62.3% of all functions observed. The three dominate metabolism functions included carbohydrate metabolism (relative abundance of 14.1–15.8%), amino acid metabolism (10.1–10.7%) and metabolism of cofactors and vitamins (6.3–7.6%), while lipid metabolism (3.3–3.7%) and glycan metabolism (2.8–4.2%) were also consistently observed in similar relative abundances. The high association of taxa associated with amino acid metabolism with BSFL suggests that the gut community could help to convert protein to amino acids that could be assimilated by the BSFL gut. In the mammalian gut, bacterial conversion of free amino acids into polypeptides contributes considerably to amino acid metabolism and bioavailability in the host (Dai *et al*., 2010). In the insect gut, microorganisms also contribute to nutrient provisioning to their host. For instance, *Ishikawaella capsulatus* provisions essential amino acids to plant‐feeding plataspid stinkbugs (Nikoh *et al*., 2011). It is still not clear what role the BSFL host plays in assimilation of nitrogen and/or production of amino acids and other nitrogen‐containing compounds, but the protease and trypsin‐like protease observed in the BSFL gut (Kim *et al*., 2011) may provide one interpretation, which is that the BSFL need the protease to decompose some protein to polypeptides and/or amino acids for further absorbing. Here, the intestinal microbes may provide the similar function as protease to BSFL, due to the high‐level amino acid metabolism genes. This finding may partly explain the biological mechanisms of high‐level protein accumulation in the BSFL body (Xiao *et al*., 2018) through interactions with intestinal microbes.

## Conclusion

We investigated the intestinal bacterial communities of BSFL in chicken and swine manure conversion systems and characterized the dominant microbes and predicted nutrient functions of intestinal bacteria in the gut larvae. Proteobacteria, Firmicutes and Bacteroidetes were the dominant phyla, while *Enterococcus, Providencia* and *Morganella* were the dominant genera in the BSFL gut. Changes in OM, TP, *etc*., in swine and chicken manure were associated with changes in the microbial community. Some bacteria taxa with putative nutrient‐provisioning abilities, such as *Rhizobiales*, *Burkholderia* and Bacteroidales, were also found in BSFL, which may be associated with nitrogen fixation or recycling, supplying essential amino acids, *etc*. The relative abundance of amplicons derived from *Providencia* was strongly correlated with proteins and lipids, suggesting *Providencia* might be one important population in gut to decomposing proteins and lipids. The high relative abundance of taxa associated with amino acid metabolism functions may partly explain the biological mechanisms of high‐level protein accumulation in the BSFL body through interaction with intestinal microbes. However, more work is needed to confirm these hypotheses (high protein accumulation, etc.). Taken together, the results provide valuable information for understanding the biological mechanisms of the high‐efficiency conversion of nutrients by BSFL associated with intestinal microbes. Our findings of microbial succession during BSFL waste conversion, along with changes in nutrient cycling within the manure, demonstrate that gut bacteria likely play an important role in the complex waste conversion process.

## Experimental procedures

### Insect and livestock manure sources

Black soldier fly larvae were obtained from a colony maintained throughout the year in Huazhong Agricultural University, Wuhan, China (Zheng *et al*., 2013; Li *et al*., 2015). The breeding systems were in a greenhouse at 27.5°C with 70% relative humidity. The larvae were fed with a mixture of wheat flour and bran every two days, and the adult flies were collected in the cages (1.2 m × 1.0 m × 1.5 m) under natural sunshine for mating and laying eggs. Six‐day‐old larvae were collected from the colonies and used in the experiments. The swine and chicken manure were obtained from Chaotuo Ecological Agriculture, Wuhan City, China.

### Livestock manure conversion systems and sampling

Swine (or chicken) manure (10 kg) was stored in plastic containers (60 cm length, 40 cm width and 10 cm height) with three replicates in each group. Approximately 10 000 6‐day‐old BSFL were added to each transformation group. All experiments were carried out in a greenhouse at 27.5°C with 70% relative humidity. Conversion lasted for 12 days. Manure samples (50 g) and 10 larvae were randomly and aseptically collected every 4 days. Manure samples were freeze‐dried aseptically and stored at −80°C for further chemical analysis.

### Extraction of total DNA from BSFL guts

The sampled larvae were starved for 24 h to empty their gut to reduce the feed and excrement with non‐symbiotic bacteria. The surface impurities of the BSFL were washed off with sterile water, immersed in 75% ethanol for 3 min and then rinsed three times with sterile water (Crippen and Sheffield, 2006). Thereafter, the whole guts of five larvae were sterilely dissected, pooled, ground and stored at −80°C for community analysis of the intestinal microbiota. Cells were disrupted using CTAB, which also removed complex carbohydrates and proteins from the mixture. The phenol–chloroform method was used for DNA extraction (Zhou *et al*., 1996).

### Pyrosequencing of bacterial 16S rRNA genes

The DNA samples were analysed by sequencing the V3‐V4 regions of bacterial 16S rRNA (Majorbio Bio‐Pharm Technology, Shanghai, China) for community profiling of the intestinal microbiotas. The primers used were 338F (5ʹ‐actcctacgggaggcagcag‐3ʹ) and 806R (5ʹ‐ggactachvgggtwtctaat‐3ʹ). The PCRs were conducted using the following program: 3 min of denaturation at 95°C, 27 cycles of 30 s at 95°C, 30 s for annealing at 55°C and 45 s for elongation at 72°C, and a final extension at 72°C for 10 min. PCRs were performed in technical triplicate 20 μl mixtures containing 4 μl of 5× FastPfu Buffer, 2 μl of 2.5 mM dNTPs, 0.8 μl of each primer (5 μM), 0.4 μl of FastPfu Polymerase and 10 ng of template DNA. The resulting PCR products were extracted from a 2% agarose gel and further purified using the AxyPrep DNA Gel Extraction Kit (Axygen Biosciences, Union City, CA, USA) and quantified using QuantiFluor™‐ST (Promega, USA) according to the manufacturer’s protocol. Purified amplicons were pooled in equimolar concentrations and sequenced on an Illumina MiSeq platform (Illumina, San Diego, CA, USA) in PE300 mode according to the standard protocols provided by Majorbio Bio‐Pharm Technology. Raw fastq files were demultiplexed, quality‐filtered by Trimmomatic and merged by FLASH with the following criteria: (i) the reads were truncated at any site receiving an average quality score < 20 over a 50 bp sliding window. (ii) Primers were exactly matched allowing 2 nucleotide mismatching, and reads containing ambiguous bases were removed. (iii) Sequences whose overlap longer than 10 bp were merged according to their overlap sequence. The read counts of 21 samples have been normalized to the minimum count (50 781) before the OTU analysis. Operational taxonomic units (OTUs) were clustered with a 97% similarity cut‐off using UPARSE (version 7.1; http://drive5.com/uparse/), and chimeric sequences were identified and removed using UCHIME. The taxonomy of each 16S rRNA gene sequence was analysed by the RDP Classifier algorithm (http://rdp.cme.msu.edu/) against the SILVA (SSU123) 16S rRNA database using a confidence threshold of 70%.

The functions of the gut microbes of BSFL in chicken and swine manure were determined by using ‘Tax4Fun’ based on the SILVA database. Tax4Fun converted the 16S taxonomies to the prokaryotic taxonomies for the KEGG database (Kyoto Encyclopedia of Genes and Genomes). Then, the description and functional information of each prokaryote could be obtained from the KEGG database (http://www.genome.jp/kegg/).

### Analyses of nutrient elements

To explore the relationship between dynamic changes in intestinal microflora and the nutrients of the samples, we selected organic matter (OM), total nitrogen (TN), total phosphorus (TP), total potassium (TK) and crude fat (CF) as nutrient elements. The concentrations of TN, TP, TK and OM in the manure samples were assayed according to standard methods specified in NY 525‐2012 Standardization Administration of the People’s Republic of China; CF was assayed by Soxhlet extraction (Virot *et al*., 2007).

### Statistical methods

The statistical analyses were conducted by using the R package software and Microsoft Office 365 (Excel, version 16.16.18). Community richness parameters, community diversity parameters and a sequencing depth index were calculated by using the mothur software. Significance was determined at the *P* < 0.05 and 95% confidence level by Tukey test.

## Conflict of interests

The authors declare no competing interests.

## Supporting information


**Fig. S1.** Rarefaction curves of the 16S rRNA gene reads based on OTUs at 97% sequence similarity.
**Fig. S2** **.** The relative abundance of phylum‐level taxa for 21 intestinal bacterial samples of BSFL in chicken manure (CHT) and swine manure (SWT) treatment systems. D0, for the initial larval gut; CHT/SWT 4/8/12, for the larval gut from manure system in the 4th/8th/12th day.
**Table S1** **.** The details of sequence determine information for the 21 samples.
