# Supplementary material for: Characteristics and nutrient function of intestinal bacterial communities in black soldier fly (Hermetia illucens L.) larvae in livestock manure conversion
Source: Microb Biotechnol. 2020 May 25;14(3):886–96. doi: 10.1111/1751-7915.13595 (PMC8085981; doi:10.1111/1751-7915.13595)
Supplement: Supplementary file 1 — Fig. S1. Rarefaction curves of the 16S rRNA gene reads based on OTUs at 97% sequence similarity. Fig. S2 . The relative abundance of phylum‐level taxa for 21 intestinal bacterial samples of BSFL in chicken manure (CHT) and swine manure (SWT) treatment systems. D0, for the initial larval gut; CHT/SWT 4/8/12, for the larval gut from manure system in the 4th/8th/12th day. Table S1 . The details of sequence determine information for the 21 samples. [file MBT2-14-886-s001.docx]

**Supplement Materials**


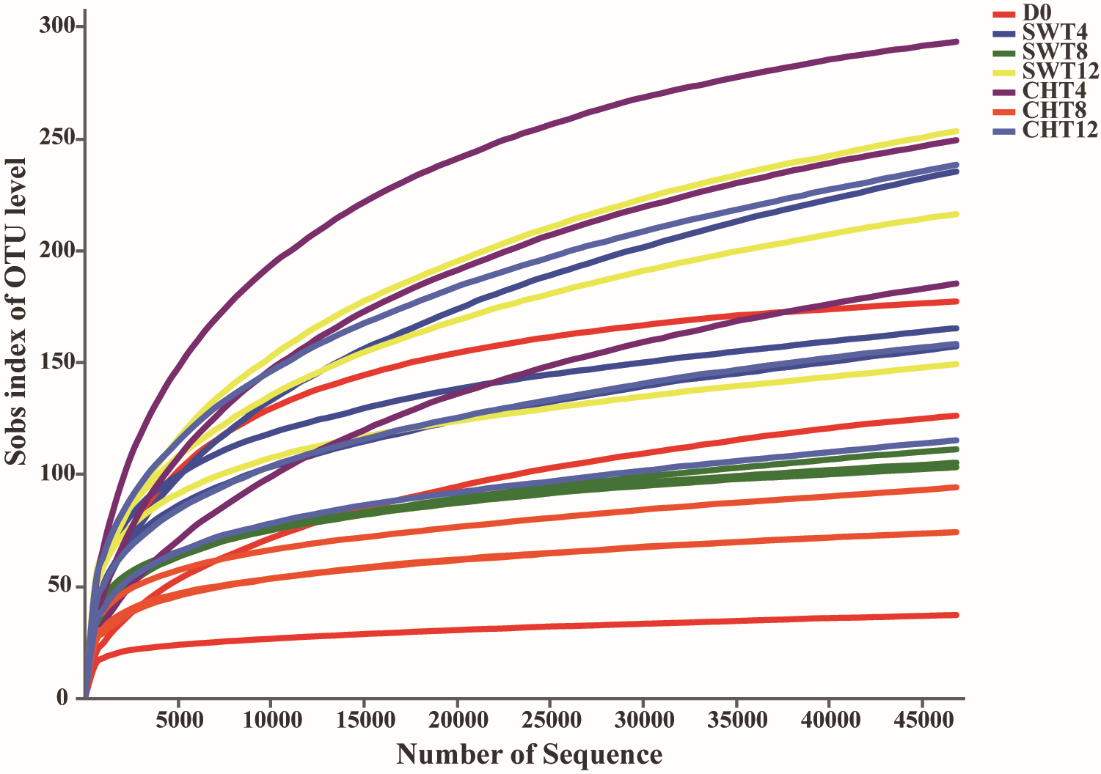


**Fig. S1 Rarefaction curves of the 16S rRNA gene reads based on OTUs at 97% sequence similarity.**


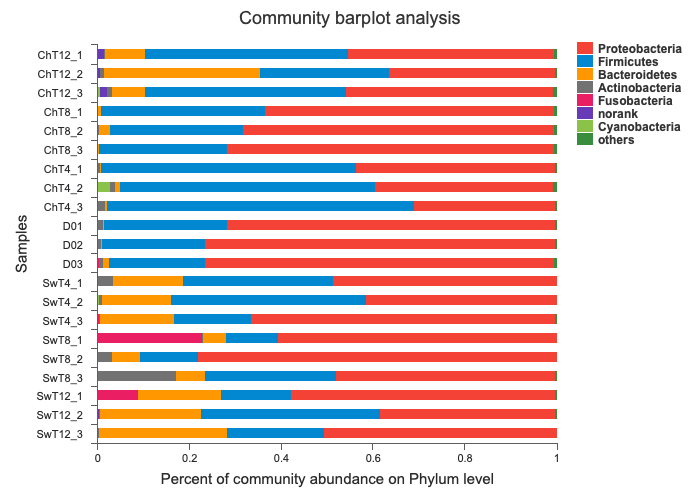


**Fig. S2 The relative abundance of phylum-level taxa for 21 intestinal bacterial samples of BSFL in chicken manure (CHT) and swine manure (SWT) treatment systems. D0, for the initial larval gut; CHT/SWT 4/8/12, for the larval gut from manure system in the 4^th^/8^th^/12^th^ day.**

**Table S1 the details of sequence determine information for the 21 samples**

| **Sample\Info** | **Seq_num** | **Base_num** | **Mean_length** | **Min_length** | **Max_length** |
| --- | --- | --- | --- | --- | --- |
| D01 | 50781 | 22830641 | 449.5902 | 359 | 498 |
| D02 | 56251 | 25307855 | 449.9094 | 413 | 453 |
| D03 | 53032 | 23801330 | 448.8107 | 273 | 477 |
| SwT4_1 | 58061 | 25649513 | 441.7684 | 276 | 453 |
| SwT4_2 | 62020 | 27656536 | 445.9293 | 367 | 455 |
| SwT4_3 | 58040 | 25915233 | 446.5064 | 308 | 453 |
| SwT8_1 | 61847 | 27467983 | 444.128 | 307 | 452 |
| SwT8_2 | 55049 | 24661882 | 447.9987 | 293 | 460 |
| SwT8_3 | 61577 | 27555323 | 447.4938 | 366 | 460 |
| SwT12_1 | 57854 | 25650716 | 443.3698 | 300 | 467 |
| SwT12_2 | 63214 | 27793876 | 439.6791 | 266 | 454 |
| SwT12_3 | 55241 | 24212525 | 438.3071 | 287 | 469 |
| ChT4_1 | 52871 | 23758106 | 449.3599 | 294 | 453 |
| ChT4_2 | 63340 | 28316737 | 447.0593 | 275 | 477 |
| ChT4_3 | 55843 | 25051922 | 448.6135 | 271 | 452 |
| ChT8_1 | 62755 | 27873201 | 444.159 | 366 | 462 |
| ChT8_2 | 55235 | 24777419 | 448.5819 | 346 | 452 |
| ChT8_3 | 63441 | 28195422 | 444.4353 | 366 | 476 |
| ChT12_1 | 61326 | 27540989 | 449.0916 | 303 | 455 |
| ChT12_2 | 61033 | 27317807 | 447.5908 | 346 | 467 |
| ChT12_3 | 55928 | 25072851 | 448.3059 | 271 | 496 |
